# Multiple chemical sensitivity described in the Danish general population: Cohort characteristics and the importance of screening for functional somatic syndrome comorbidity—The DanFunD study

**DOI:** 10.1371/journal.pone.0246461

**Published:** 2021-02-24

**Authors:** Thomas Meinertz Dantoft, Steven Nordin, Linus Andersson, Marie Weinreich Petersen, Sine Skovbjerg, Torben Jørgensen

**Affiliations:** 1 Center for Clinical Research and Prevention, Bispebjerg & Frederiksberg Hospital, Frederiksberg, Capital Region Denmark; 2 Department of Psychology, Umeå University, Umeå, Sweden; 3 The Research Clinic for Functional Disorders and Psychosomatics, Aarhus University Hospital, Aarhus, Denmark; 4 Department of Clinical Medicine, Danish Center for Mindfulness, Aarhus University, Aarhus, Denmark; 5 Department of Public Health, Faculty of Health and Medical Sciences, University of Copenhagen, Copenhagen, Denmark; 6 Faculty of Medicine, Aalborg University, Aalborg, Denmark; Shahjalal University of Science and Technology, BANGLADESH

## Abstract

**Background:**

Multiple chemical sensitivity (MCS) is characterized by widespread symptoms attributed to exposure to airborne chemicals. MCS is categorized as a functional somatic syndrome (FSS), and MCS cases often meet the criteria for other types of FSS, e.g. fibromyalgia. The primary aim was to characterize MCS regarding symptom triggers, symptoms, lifestyle and describe demographics, socioeconomics and lifestyle factors associated with MCS. A secondary aim was to examine the implication of FSS comorbidity.

**Methods:**

Data were derived from a random sample of the Danish adult population enrolled in the Danish Study of Functional Disorders (DanFunD; n = 9,656). Questionnaire data comprised information used to delimit MCS and four additional types of FSS, as well as data on demographics, socioeconomics and lifestyle. MCS cases (n = 188) was stratified into subgroups; MCS only (n = 109) and MCS with comorbid FSS (n = 73). Information regarding FSS comorbidities were missing for six MCS cases. MCS subgroups and controls without FSS comorbidities (n = 7,791) were compared by means of logistic regression analyses, adjusted for age and sex.

**Results:**

MCS was associated with female sex, not being in occupation and low social status, but not with age or education. MCS cases reported normal dietary intake and smoking habits and lower alcohol consumption. Additional associations were found between MCS and low rate of cohabitation, sedentarism, daily physically limitations, and poor quality of sleep. However, subgroup analysis revealed that these findings were primarily associated with MCS with comorbid FSS.

**Conclusions:**

MCS was associated with lower socioeconomic status, physically inactivity and poor quality of sleep. Subgroup analysis revealed that several associations was explained by FSS comorbidity, i.e. MCS cases with no comorbid FSS showed normal rate of cohabitation and did not report physical limitations or difficulties sleeping. Overall, our findings emphasise the importance of screening MCS cases for FSS comorbidity both in epidemiological and clinical settings.

## Introduction

Most people are on a regular basis exposed to a range of airborne chemicals that they may notice, but do not pay much attention to. Such exposures can be perfumed products, car exhaust, organic solvents and cooking fumes as some common examples [1,2]. It is also common to have experienced being bothered by such exposures to a degree that may be regarded as unpleasant or even be the cause of transient somatic discomfort [2,3]. However, for some individuals, these common airborne chemical exposures that most people consider to be benign, are associated with recurrent disabling physical reactions [4–6]. This condition, by the patients attributed to an acquired increase in sensitivity to airborne chemical, is often referred to as multiple chemical sensitivity (MCS). MCS patients constitute a highly heterogeneous group in term of symptom pattern and severity, level of sensitivity and the nature of exposures associated with symptom elicitation. Exposure agents associated with MCS symptoms are numerous and chemically diverse, and individuals afflicted with MCS often describe that symptoms appear when exposed to substances that carry an odour [5]. A traditional toxicological dose-response relationship between exposure levels and elicitation of symptoms or symptom severity does not exist for MCS [7,8], and some cases even report symptom elicitation upon an exposure that is below their sensory detection threshold [9]. The type and number of symptoms experienced by persons with MCS are likewise diverse, with common symptoms being severe headache/migraine and dizziness, respiratory symptoms, muscle and joint and pain [4,10–12].

Numerous and very diverse modes of action have been suggested to explain the MCS phenotype, with some of the more commonly proposed causal mechanisms being central pain sensitization, neurogenic inflammation, altered metabolic capacity, behavioural conditioning, and expectancy-induced nocebo effect [5,9,13–15]. However, scientific evidence supporting the suggested mode of action is still insufficient to reach consensus, and the underlying mechanisms leading to symptom elicitation in MCS remains an enigma. It is therefore a challenge how best to delimit MCS, and in the literature MCS case status is either determined based on self-reports (i.e. self-reported clinician diagnosed MCS [9]), self-assessment using validated questionnaire instruments [3,5,16,17]) or diagnoses by an experienced physician [18].

Consequently, reported prevalence rates of MCS vary considerable in the literature, with most estimates ranging from 0.5%-6.5% [2,12,19–21]. Likewise, a number of alternative labels are used in the literature to describe the same phenotype with chemical intolerance, environmental hypersensitivity, toxicant induced loss of tolerance and idiopathic environmental intolerances being some of the most commonly applied labels [7,22–25]. Presently, we use MCS without reference to any assumptions about the underlying etiology or causation.

MCS is currently categorized as a functional somatic syndrome (FSS), and MCS cases often meet the criteria for FSS subtypes such as fibromyalgia (FM), chronic fatigue syndrome (CFS) and irritable bowel syndrome (IBS) [5,26,27]. This symptomatic overlap between MCS and other types of FSSs represents a challenge when studying the epidemiology of MCS, and no consensus exists on how best to handle this potential influence of comorbid FSS. In some studies of MCS, cases fulfilling criteria for other types of FSSs are not identified, thereby ignoring the issue of comorbid FSS, whereas in others studies, cases with comorbid FSS are identified and excluded from the population in order to study a more “pure” form of MCS [17]. The later procedure was even recommended by Lacour and colleagues, but only for cases in which comorbid FSS had emerged before the development of MCS [22]. However, whether a strategy of excluding MCS cases with FSS comorbidity is scientifically sensible or whether it in fact contributes to a more selected and less representative study population is a matter of discussion.

So far, the epidemiology of MCS has predominantly been studied in clinical and occupational settings, and less in randomly selected general population-based samples [28]. General population-based cohort studies have played a significant role in unravelling the epidemiology of many chronic diseases [29,30], and has the advantages of being less prone to selection bias, provide reliable prevalence estimates, and can describe the natural history of a disease [31,32]. These methodological advantages advocate for a prioritization of more general population-based research when studying a disease such as MCS, for which both prevalence and aetiology remains poorly described [5,28,33,34]. The Danish Study of Functional Disorders (DanFunD) was initiated in 2011 to outline the epidemiology of FSSs and represents a general population-based longitudinal cohort study of nearly 10,000 adult Danes. In the DanFunD study, participants fulfilling criteria for MCS, FM, IBS, CFS and whiplash-associated disorders (WAD) were identified using standardized validated questionnaire-based delimitations [35]. These five types of FSS were chosen to represent the field of FSS, but other types of FSS could have been included instead, such as temporomandibular disorder, tension-type headache or pelvic pain syndromes. The results from the DanFunD study have shown that FSSs are prevalent in the general population and that a high level of mutual overlap between the five FSSs examined exist [26,36].

Using an epidemiological approach and data from the DanFunD study, the primary aim of this study was to characterize MCS phenotype regarding symptom triggers, symptom experienced and adjustment in lifestyle as well as describe the demographics, socioeconomic status and selected lifestyle factors associated with MCS. A secondary aim was to investigate whether MCS cases without comorbid FSS differs from MCS cases with comorbid FSS in the examined parameters.

## Methods

### Study population

The study population comprised a random sample of 29,088 persons drawn from the Danish Civil Registration System. A total of 9,656 (33.7%) accepted the invitation and participated in the DanFunD baseline examination conducted between 2011 and 2015 [35]. All participants were Danish citizens born in Denmark aged 18–76 years and living in one of 10 selected municipalities covering the western suburbs of the greater Copenhagen area, Denmark.

### Case definition

Questionnaire data from DanFunD comprises information used to delimit five different FSSs in the cohort, i.e. MCS [22,37], FM [38], CFS [39,40], IBS [41] and WAD [42] as described in more detail by Petersen and colleagues [26]. Case criteria for MCS were constructed as an abridged adaptation of the 1999 US Consensus Criteria for MCS and the revisions suggested by Lacour and colleagues [22,37]. Hence, MCS case status was assigned to participants reporting widespread impairing symptoms and lifestyle adjustments as a result of their reactions to common airborne exposures (S1–S3 Tables), operationalized as follows

1. Have experienced symptoms upon being exposed to at least two of 11 common odours and airborne chemicals, and
2. have experienced at least one symptom from the central nervous system and at least one symptom from another organ system in response to inhalation of airborne odours or chemicals, and
3. Report significant lifestyle or functional impairment due to symptoms related to inhalation of airborne chemicals and odour, defined as; responding affirmably to: A) symptoms have influenced my choice of products used for personal hygiene, products used for cleaning at home, and/or choice of places where I do my daily shopping, and either B) symptoms have negatively influenced my social lifestyle (i.e. limited my possibility to use of public transport, dine at restaurants, go to the cinema/theatre, participate in meetings/social events) or C) symptoms have negatively influenced my occupational conditions (i.e. have had to go on temporary sick leave, discontinued education/employment, been unable to hold a job position or unable to complete an education).

All participants fulling criteria for MCS were subsequently divided into two subgroups, i.e. one subgroup with MCS cases with no comorbid FSS, and one subgroup with MCS cases fulfilling the criteria for at least one of the additional four FSS screened for in the DanFunD study.

#### Demographics and socioeconomic status

For descriptive analysis, participants were stratified into five age bands of 10 years (except for the most senior group covering 60–76 years of age). Cohabitation was divided into three categories married/cohabiting, formerly married/cohabited and never married/cohabited. Educational level was stratified into four categories beyond elementary school, i.e. skilled worker or <1 year of higher education, short vocational training/<3 years, medium length vocational training/3-4 years, and long vocational training/> 4 years. Occupational status comprised the three categories currently in occupation, formerly in occupation, and never having been in occupation. Participants were also asked to rate their subjective social status on a visual analogue scale ranging from one (lowest social status) to ten (highest social status). For analysis, the ratings were divided into three categories, i.e. those with low (1–4), middle (5–7) or high (8–10) subjective social status [43].

#### Lifestyle factors

Smoking was stratified into four categories (smoking daily, smoking occasionally, ex-smoker, never smoked regularly), dietary intake was estimated using a self-administered 26-item food frequency questionnaire, and stratified into three levels of diet (healthy, average, unhealthy) [44], weekly alcohol consumption was stratified into four categories (zero, 1–21 (men)/1-14 (women), 21–35 (men)/14-35 (women), ≥36 units/week), physical activity during leisure time was stratified into three categories (sedentary, low activity and medium/high activity) [45], being limited in daily activities was stratified into three categories (all the time/frequently, sometimes and almost never/never), problems falling asleep at night was stratified into three frequencies of difficulties falling asleep (2–4 times a month or less, 1–6 times a week, every night), and waking up earlier in the morning than anticipated was stratified into three frequencies of difficulties falling asleep (2–4 times a month or less, 1 or more times a week, every morning).

### Statistical analyses

After completion of the data collection for the DanFunD study, a thorough inspection of all collected data was conducted by experienced statisticians. All measures were checked using standardised templates for handling of missing data and error values. In general, rates of missing data in the DanFunD study are low, and in this specific study the rate is below 5%, and for FSS below 3%.

**Fig 1:**
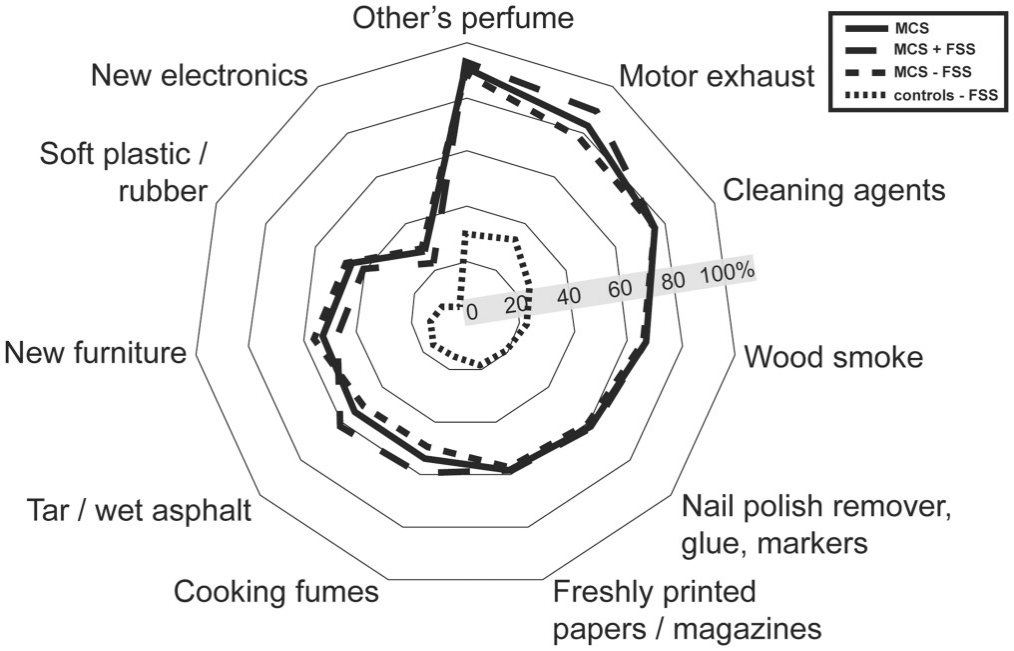
Prevalence of participants that have experienced unpleasant reactions when exposed to any of these 11 types of common odours or airborne chemicals. Abbreviations: MCS = Multiple chemical sensitivity; FSS = functional somatic syndrome; MCS all = all participants meeting criteria for multiple chemical sensitivity; MCS + FSS; participants meeting criteria for multiple chemical sensitivity and one or more comorbid FSS. MCS ÷ FSS = participants meeting criteria for multiple chemical sensitivity but not comorbid FSS.

**Fig 2:**
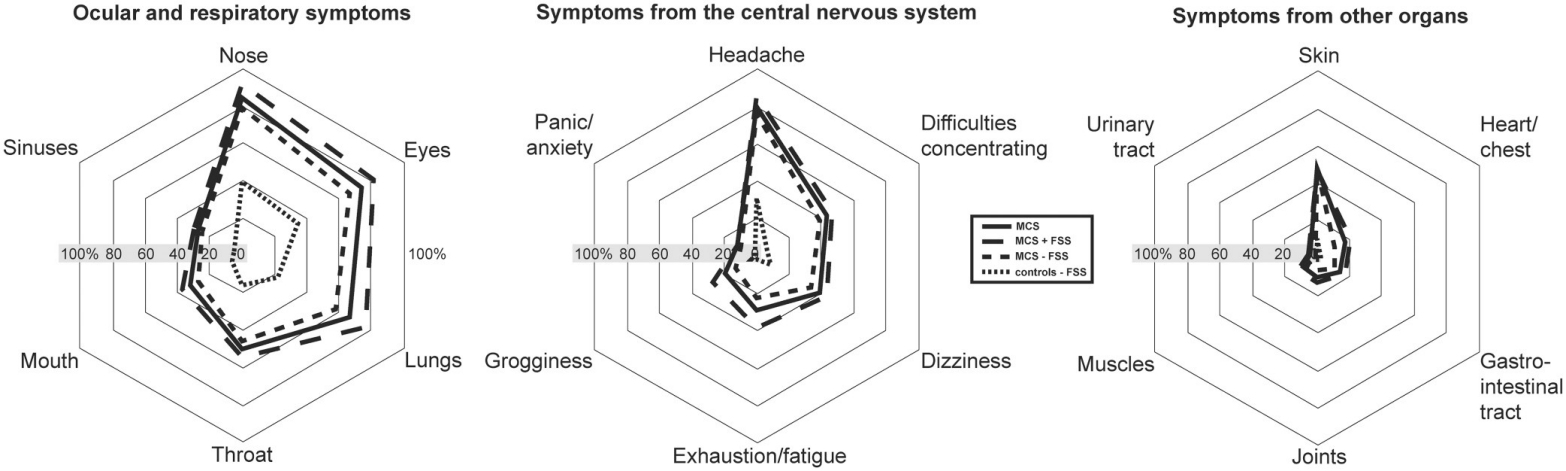
Prevalence of symptoms associated with inhalation of airborne chemicals. Abbreviations: MCS = Multiple chemical sensitivity; FSS = functional somatic syndromes; MCS all = all participants meeting criteria for multiple chemical sensitivity; MCS + FSS = participants meeting criteria for multiple chemical sensitivity and one or more comorbid FSS. MCS ÷ FSS = participants meeting criteria for multiple chemical sensitivity but not comorbid FSS.

**Fig 3:**
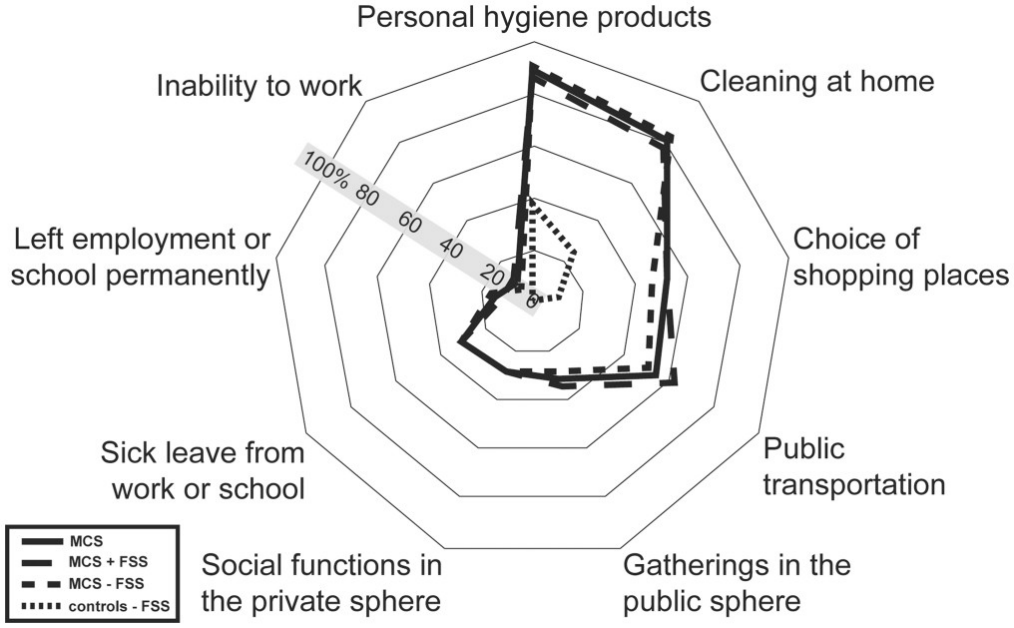
Prevalence of participants reporting adjustments of behaviour due to symptoms related to inhalation of airborne chemicals. Abbreviations: MCS = Multiple chemical sensitivity; FSS = functional somatic syndromes; MCS all = all participants meeting criteria for multiple chemical sensitivity; MCS + FSS; participants meeting criteria for multiple chemical sensitivity and one or more comorbid FSS. MCS ÷ FSS = participants meeting criteria for multiple chemical sensitivity but not comorbid FSS.

Statistical analyses were performed using SPSS for Windows (version 22; IBM Corp, Armonk, NY). All p-values were two-sided, and p-values ˂0.05 were considered statistically significant. Participants were divided into four groups: 1) MCS all = all cases fulfilling criteria for MCS; 2) MCS ÷ FSS = cases fulfilling criteria for MCS, but not for any of the four additional FSS in the DanFunD study; 3) MCS + FSS = Cases fulfilling criteria for MCS and at least one other FSS; and 4) Controls = not fulling criteria for any of the five FSS examined. Descriptive statistics for the four groups are presented as % (n) or mean (standard deviation, SD). Each of the three MCS groups were compared to the control group, and the MCS ÷ FSS subgroup was compared with the MCS + FSS subgroup. For all group comparisons, Pearson Chi-squared tests were used for all categorical variables, i.e. data used to delimit MCS (Fig 1/S1 Table; Fig 2/S2 Table; Fig 3/S3 Table), and demographic, socioeconomic and lifestyle data. Independent sample t-test was used for numerical variables (age as a continues variable). By means of logistic regression analysis, sex as a five class categorial variable was adjusted for age, whereas data on chemical hypersensitivity, socioeconomics and lifestyle parameters were adjusted for age and sex. Sex as a continuous variable was adjusted for age by means of simple linear regression analysis [26,46].

#### Ethical approval

All research reported here was conducted in accordance with the Helsinki Declaration. All participants gave written informed consent before participating, and the study was approved by the Ethical Committee of Copenhagen County (Ethics Committee: KA-2006-0011; H-3-2011-081; H-3-2012-0015) and the Danish Data Protection Agency.

## Results

### Functional somatic syndromes

A total of 188 (1.95%) persons in the DanFunD cohort fulfilled the criteria for MCS, whereas 7,971 (82.5%) did not fulfil criteria for any of the five FSSs in question (Table 1). Of the 188 identified MCS cases, 109 (1.1% of the cohort/61.2% of all MCS cases) only fulfilled criteria for MCS, and were assigned to the MCS with no comorbid FSS subgroup, and 73 cases (0.8% of cohort/38.8% of all MCS cases) fulfilled the criteria for one or several of the additional four FSSs, and were assigned to the MCS with comorbid FSS subgroup. Most common FSS comorbidity among the MCS cases were CFS (51 cases) followed by FM (30 cases), IBS (19 cases) and WAD (9 cases). Six MCS cases could not be assigned to either of the two MCS subgroups due to missing questionnaire data on comorbid FSS status. Hence, those cases were not included in the comparison of MCS cases with and without comorbid FSS. Cases with MCS were more often females, independently of FSS comorbidity, whereas age did not differ between MCS cases and controls or between the MCS cases with or without comorbid FSS (Table 1).

**Table 1:** Demographic characteristics of participants in the DanFunD study fulfilling criteria for multiple chemical sensitivity (MCS) and of controls not fulling the criteria for any of five functional somatic syndromes (FSSs).

	MCS all (n = 188)	MCS + FSS comorbidity (n = 73)	MCS ÷ FSS comorbidity (n = 109)	Controls ÷ FSS (n = 7791)
**Sex % (n)**				
Men	33.0 (62)*	27.4 (20)*	38.5 (42)*	48.9 (3812)
Women	67.0 (126)*	72.6 (53)*	61.5 (37)*	51.1 (3979)
**Age, mean (SD)** †	53.6 (13.5)	51.3 (13.4)	54.8 (13.8)	52.7 (13.1)
Age % (n)**				
18–29	8.5 (16)	9.6 (7)	8.3 (9)	7.3 (572)
30–39	9.6 (18)	13.7 (10)	7.3 (8)	9.1 (706)
40–49	14.9 (28)	16.4 (12)	12.8 (14)	21.2 (1650)
50–59	26.1 (49)	27.4 (20)	25.7 (28)	25.1 (2018)
60–76	41.0 (77)	32.9 (24)	45.8 (50)	36.5 (2845)

Standard deviation (SD).

MCS all: All participants meeting criteria for MCS. MCS + FSS: Participants meeting criteria for MCS and one or more comorbid FSS. MCS **÷** FSS: Participants meeting criteria for MCS but not comorbid FSS.

*Pearson Chi-square test comparing MCS groups with controls and MCS subgroups with each other (p<0.05), adjusted for age.

** Pearson Chi-square test comparing MCS groups with controls and MCS subgroups with each other (p<0.05), no significant differences.

^†^Independent samples t-test comparing MCS groups with controls (p<0.05), no significant differences.

### Airborne chemical exposure related to unpleasant reactions

All questions used to delimit MCS (S1–S3 Tables) were also found to be positively associated with MCS case status (except for the MCS ÷ FSS group not being associated with urinary tract symptoms). Of the 11 types of chemical exposure included in the questionnaire (Fig 1, S1 Table), other persons use of perfumed products, motor vehicle exhaust and cleaning agents were the exposure types most frequently reported to be the cause of unpleasant reactions upon inhalation, both among controls and MCS cases (Fig 1; S1 Table). Reports of unpleasant reactions caused by inhalation of motor vehicle exhaust were more common among MCS cases with comorbid FSS than MCS cases with no co-morbid FSS (Fig 1; S1 Table).

### Symptoms related to inhalation of airborne chemicals

The most common symptoms reported by the MCS cases and controls upon inhalation of airborne chemicals and odours (Fig 2; S2 Table) were symptoms from the ocular and respiratory system and headaches from the central nervous system (Fig 2; S2 Table). Symptoms from the nose, eyes, lungs, exhaustion/fatigue, grogginess and heart/chest were more frequently reported by MCS cases with comorbid FSS compared to MCS cases with no comorbid FSS (Fig 2; S2 Table).

### Consequences in daily life related to inhalation of airborne chemicals

Nearly 90% of the MCS cases and 39% of the controls reported that symptoms related to inhalation of odours and airborne chemicals have influenced their choice of products used for personal hygiene, and 80% of the MCS cases and 25% of the controls had made changes related to the choice of cleaning products used domestically (Fig 3, S3 Table). Furthermore, 53% of the MCS cases and 10% of the controls reported that symptoms associated with odours and airborne chemicals had affected their shopping habits. However, only MCS cases reported that symptoms related to inhalation of airborne chemicals had affected their social life or occupational conditions (Fig 3, S3 Table).

### Demographics and socioeconomics

A larger percentage of the MCS cases with comorbid FSS were living alone compared to both the MCS cases without comorbid FSS and the controls, whereas educational level for all MCS cases were comparable to controls (Table 2). Fewer MCS cases were in current occupation compared to the controls, and the MCS cases considered their own position in society to be lower than reported by controls, independently of FSS comorbidity (Table 2). Regarding subjective social status, no group differences was observed for the middle group to which most cohort participants belonged too, whereas the MCS cases were more likely to consider themselves as having low socioeconomic position and less likely to view it to be high compared to controls.

**Table 2:** Socioeconomic status of participants meeting criteria for multiple chemical sensitivity (MCS) and of controls not meeting the criteria for any of five functional somatic syndromes (FSS).

	MCS all (n = 188)	MCS + FSS comorbidity (n = 73)	MCS ÷ FSS comorbidity (n = 109)	Controls ÷ FSS (n = 7791)
**Cohabiting % (n)**		*	†	
Married/cohabiting	75.4 (141)	66.7 (48)	80.7 (88)	78.7 (6116)
Formerly married/cohabiting	20.3 (38)	29.2 (21)	14.7 (16)	15.1 (1173)
Never married/cohabiting	4.3 (8)	4.2 (3)	4.6 (5)	6.2 (478)
**Education % (n)**				
Skilled worker or < 1-year higher education after school	41.2 (63)	39.2 (20)	43.3 (42)	42.8 (3245)
< 3 years higher education	20.3 (31)	21.6 (11)	17.5 (17)	19.2 (1453)
3- or 4-years higher education	31.4 (48)	33.3 (17)	30.9 (30)	25.8 (1959)
> 4 years higher education	7.2 (11)	5.9 (3)	8.2 (8)	12.2 (929)
**Occupational status % (n)**	*	*	*	
Currently in occupation	54.6 (100)	50.7 (35)	58.3 (63)	69.6 (5372)
Formerly in occupation	44.3 (81)	46.4 (32)	41.7 (45)	29.1 (2249)
Have never been in occupation	1.1 (2)	2 (2.9)	0.0 (0)	1.3 (98)
**Subjective social status % (n)**	*	*	*	
Low	9.7 (18)	12.5 (9)	7.5 (8)	3.6 (278)
Middle	64.9 (120)	66.7 (48)	65.4 (70)	65.6 (5062)
High	25.4 (47)	20.8 (15)	27.1 (29)	30.8 (2378)

MCS all: All participants meeting criteria for MCS. MCS+ FSS: Participants meeting criteria for MCS and one or more comorbid FSS. MCS ÷ FSS: Participants meeting criteria for MCS but not comorbid FSS.

*Pearson Chi-square test comparing MCS groups with controls (p<0.05), adjusted for sex and age.

^†^ Pearson Chi-square test comparing MCS ÷ FSS comorbidity with MCS + FSS comorbidity, adjusted for sex and age.

### Lifestyle

Regarding lifestyle measures (Table 3), the MCS cases reported dietary and smoking habits that were comparable to those of the controls, as well as comparable level of alcohol consumption. These findings were independent of comorbid FSS status, expect for the MCS cases with no comorbid FSS being more likely to consume more alcohol than recommend (21–35 (men)/14-35 (women) units per week) compared to the MCS cases with FSS comorbidity. MCS was associated with a more sedentary lifestyle during leisure time, but only in the MCS all group and the MCS group with comorbid FSS; not in the MCS subgroup with no FSS comorbidity. The MCS cases also considered themselves to be more limited in daily activities than the controls, and this limitation in physical capacity in daily life was more pronounced among the MCS cases with comorbid FSS. Frequent problems falling asleep at night and problems waking up earlier than anticipated in the morning was also associated with MCS all and MCS with comorbid FSS, but not with MCS cases with no comorbid FSS.

**Table 3:** Selected lifestyle factors of participants meeting criteria for multiple chemical sensitivity (MCS) and of controls not meeting the criteria for any of five functional somatic syndromes (FSS).

	MCS all (n = 188)	MCS + FSS comorbidity (n = 73)	MCS ÷ FSS comorbidity (n = 109)	Controls ÷ FSS (n = 7791)
**Diet % (n)**				
Healthy	24.2 (45)	22.5 (16)	24.8 (27)	23.0 (1776)
Average	65.6 (122)	64.8 (46)	66.1 (72)	66.4 (5131)
Unhealthy	10.2 (19)	12.7 (9)	9.2 (10)	10.6 (822)
**Smoking % (n)**				
Daily	13.9 (26)	15.1 (11)	13.8 (15)	12,1 (943)
Occasionally	2.7 (5)	1.4 (1)	3.7 (4)	3.0 (250)
Ex-smokers	44.9 (84)	44.4 (32)	46.8 (51)	37.5 (2908)
Never been a daily smoker	38.5 (72)	38.9 (28)	35.8 (39)	47.0 (3660)
**Alcohol % (n)**	*	*	*	
Zero	17.4 (29)*	17.7 (11)*	18.0 (18)*	9.5 (707)
1–21 (men)/1-14 (women)	70.1 (117)	77.4 (48)	65.0 (65)	80.2 (5970)
21–35 (men)/14-35 (women)	9.0 (15)	1.6 (1)	13.0 (13) †	8.5 (633)
≥36 units/week	3.6 (6)	3.2 (2)	4.0 (4)	1.8 (137)
**Physical activity, leisure time % (n)**	*	*	†	
Sedentary	17.3 (32)	26.8 (19)	11.1 (12)	11.5 (886)
Low activity	56.8 (105)	54.9 (39)	57.4 (62)	55.1 (4260)
Medium/high activity	25.9 (48)	18.3 (13)	31.5 (34)	33.5 (2588)
**Limited in daily activities (such as using a bike cycle, domestic housework, moving a table) % (n)**	*	*	*†	
All the time/frequently	12.9 (24)	20.0 (27.8)	2.8 (3)	1.6 (127)
Sometimes	22.0 (41)	33.3 (24)	15.7 (17)	5.8 (448)
Almost never/never	65.1 (121)	38.9 (38.9)	81.5 (88)	92.6 (7177)
**Problems falling asleep % (n)**	*	*		
2–4 times a month or less	63.1 (118)	55.6 (40)	67.0 (73)	75.8 (5876)
1 or more times a week	33.7 (63)	38.9 (28)	31.2 (34)	22.9 (1774)
Every day	6.0 (3.2)	5.6 (4)	1.8 (2)	1.3 (103)
**Waking up earlier in the morning than anticipated % (n)**	*	*	†	
2–4 times a month or less	46.2 (86)	30.6 (22)	56.5 (61)	67.1 (5205)
1 or more times a week	34.4 (64)	37.5 (27)	33.3 (36)	25.1 (1948)
Every day	19.4 (36)	31.9 (23)	10.2 (11)	7.7 (600)

MCS all: All participants meeting criteria for MCS. MCS + FSS; participants meeting criteria for MCS and one or more comorbid FSS. MCS **÷** FSS; participants meeting criteria for MCS but not comorbid FSS.

*Pearson Chi-square test comparing MCS groups with controls (p<0.05), adjusted for sex and age.

^†^ Pearson Chi-square test comparing MCS ÷ FSS comorbidity with MCS + FSS comorbidity, adjusted for sex and age.

## Discussion

In this study we utilized data from the DanFunD study to describe socioeconomic status and lifestyle characteristics associated with MCS and explored the consequences of having MCS with FSS comorbidity. Epidemiological studies examining lifestyle characteristics of persons with MCS are few, and to our knowledge, no other population-based study has in parallel examined the impact of MCS and FSS comorbidity. We also examined the level of chemical sensitivity in the general population, and in line with earlier findings our results emphasize that being bothered and experiencing symptoms when exposed to common odours and airborne chemicals is a normal phenomenon in the general population [2,4,5,47–52]. However, the consequences associated with such exposures in terms of number of symptoms experienced and enforced lifestyle adjustments were significantly fewer in the control group, with the majority not being bothered at all. These results are in accordance with results from other general population-based studies from Denmark, Sweden, USA and Germany [2–4,12,51]. Among MCS cases, only few differences were identified between those with and those without comorbid FSS based on questions used to delimitate MCS (Figs 1–3, S1–S3 Tables). This suggests that the level of sensitivity towards everyday exposures is similar between these MCS subgroups. The only differences observed were that MCS cases with comorbid FSS reported a higher overall symptom burden and were more likely to have experienced unpleasant reactions caused by inhalation of motor vehicle exhaust.

### Demographic and socioeconomic characterisation

In line with existing literature, we found an association between MCS and female sex [5,19,53,54]. As for age, we found no differences between MCS cases and controls. This is comparable to the findings from two recent studies of a Finnish population-based cohort [53] and a Canadian clinical cohort [54], but differs from that found in recent Swedish and Finnish population-based cohorts, in which chemical intolerance was found to be associated with high age [53]. Regarding socioeconomic status, we found that education was not associated with MCS, and that only MCS cases with comorbid FSS were more likely to be living alone. Furthermore, we found that MCS cases were less likely to be in current occupation, and that they reported lower subjective social status than controls. These educational and occupational characteristics are similar to previous studies of both MCS [2,55–57] and other types of FSS [58,59], and it has been suggested that the typical MCS patient is a middle-aged well-educated female [56,60,61]. However, the socioeconomic characteristics presented in the literature is not consistent. For example, Kreutzer and colleagues (1999) found that neither marital status, employment nor education were predictive of MCS in a telephone administrated survey of about 4,000 subjects, representing the general population. They proposed that the inconsistencies observed concerning socioeconomic status throughout the literature are likely a consequence of the various delimitations of MCS/chemical intolerance applied [19]. Based on the cross-sectional data presented in this manuscript, it is not possible to conclude whether lower socioeconomic position and reduced labour marked attachment preceded the progression of MCS or whether it is merely a consequence of the various limitations found to be associated with MCS. However, since educational level of the MCS cases in DanFunD were found to be comparable to that of controls, and that decreased levels of cohabitation was only associated with MCS with comorbid FSS, our data may support the latter.

### Lifestyle characteristics

Regarding lifestyle factors, we found no difference between participants with MCS and the controls in regard to daily dietary intake, smoking habits or alcohol consumption, except for a higher percentage of MCS cases not consuming any alcohol (Table 3). How these findings coincide with the literature is uncertain, as studies examining daily dietary habits and nutrient intakes among persons with MCS are surprisingly rare. Instead, more studies of MCS have investigated the potential positive effect of implementing various forms of specialized nutritional/dietary intervention programs, such as diverse intake of nutritional supplement and exclusion or rotation diet, primarily is smaller case-control stetting [9,34,62,63]. However, our findings are in line with that reported from studies of associated diseases, in which daily intake of calories or overall dietary habits have been comparable between FSS cases and healthy controls [64,65]. Several previous studies of MCS have also found lower rate of daily smokers among MCS subjects, and it has been suggested that persons with MCS often avoid being exposed to exogenous chemicals, such as motor vehicle exhaust and tobacco smoke [54,57,66,67]. However, findings on alcohol consumption or smoking frequency in MCS subjects are not consistent, and studies based on data from the Finnish Twin-cohort found that individuals reporting symptom elicitation in response to common airborne chemical exposures were more likely to be current smokers and have higher alcohol consumption than controls [68,69].

Regarding physical capacity and sleep, we found that MCS is associated with a more sedentary lifestyle, with more physical limitations in daily life and more disturbed sleep. Though, these associations were more pronounced in the subgroup of MCS cases with FSS comorbidity. Similar associations between MCS and poor quality of sleep and low physical capacity has also been reported in both clinical and population-based studies of MCS. In a recent clinical study from Spain, 27% of MCS patients reported a sedentary lifestyle and 60% rated themselves to be barely active. Furthermore, half of the patients reported their average quality of sleep as “poor or insufficient” and 38% as being of “moderate” quality [63]. Moreover, sleep disturbance has also been found to be associated with MCS in a Swedish population-based cohort [70] and in other types of FSS [71–73]. Common symptoms associated with MCS and FSS in general, such as myalgia, tenderness and fatigue, can also be caused or exacerbated by prolonged periods of nonrestorative sleep [73], why prolonged periods with poor quality of sleep may contribute to aggravation of symptom level in MCS.

### Summary and implications

In summary, we found MCS to be associated with low socioeconomic status, low physical capacity in daily life and poor quality of sleep. Moreover, our results emphasize the importance of taking comorbid FSS into account when studying MCS, as the total disease burden seems to be noticeably lower for MCS cases with no comorbid FSS. How best to handle this issue of FSS comorbidity when studying MCS is, however, open for discussion. Some studies of MCS exclude participants with comorbid FSS as an attempt to eliminate the interferences of FSS comorbidity [17,22,74]. However, this approach will undoubtedly lead to a more selective and thus less representative study population and introduce considerable risk of selection bias. On the other hand, it is important to address how FSS comorbidity may influence study findings, and if the number of cases allows for it, subgroup analysis as conducted in this study can provide a more comprehensive insight into the MCS phenotype. With smaller study samples, this strategy may not be applicable, and hence a more pragmatic approach must be applied. Consequently, whether to exclude MCS cases with comorbid FSS or not will depend on the research question and the size of the study population.

In addition to the epidemiological description of MCS, our analyses reveal that adverse reactions to everyday chemical exposure is also prevalent among controls. Almost 40% of controls reported unpleasant reactions and making adjustments in everyday life because of, seemingly, safe chemical exposures. Similar results have been published before [2,4,5,49–51,53], and consequently, we suggest two implications of this pattern: First, adverse reactions to chemicals is arguably the distinguishing criterion for defining MCS. However, reactions to scented products are so common in the general population that the line between cases and controls runs the risk of becoming blurred. It may at least partly explain the vague, heterogeneous nature of MCS, and calls for future discussions on how MCS should be defined. Second, the reactions to everyday chemicals and odours, and the related lifestyle adjustments suggest that such exposures are of concern in hypersensitive individuals in general. It may be necessary in the future to discuss these exposures in the way that is currently done for e.g. noise [75,76].

### Strengths and limitations

An important strength of this study is the large random sample of the general adult population enrolled in the DanFunD study, comprising both sexes over an age span of 50 years, and the vast amount of data collected for the study [35]. These data offer a unique opportunity within MCS research to study the epidemiology of MCS in a large and well characterized cohort, and to investigate the role of comorbid FSS. Regarding the limitations, a concern is the relatively low participation rate of the DanFunD study at 33.7%, and whether the cohort is representative of the general adult population.

A comparison between respondents and non-respondents enrolled in a comparable cohort, the Health2006 study covering the same geographical area as DanFunD, did show some differences with respect to sociodemographic characteristics, education and use of health services [77]. For example, respondents were older and better educated, and had a higher personal income than non-respondents. Other differences included larger proportion of men and more individuals living alone as well as more events of hospitalization and more days of hospitalization among the non-respondents. We expect similar differences between responders and non-responders in the DanFunD cohort, but how these differences may influence the study findings is difficult to assess. It is obviously important to take these differences into consideration when results from the DanFunD study are used to generalize on a population level. Nevertheless, we expect the selection bias to have influenced the recruitment of participants with an FSS in a similar manner, resulting in a fair representation of the general population. Hence, we believe that our results are generalizable.

The questionnaire used to delimit MCS in DanFunD was first used in the Health2006 study [77], where it was tested for linguistic comprehension, reproducibility, relevance and reliability in a pilot group setting, consisting of individuals with self-reported chemical sensitivity [2]. It was concluded that the questions have good reproducibility, and a fair concordance for both positive and negative answers [2], supporting the applicability of the questionnaire as a tool to study MCS in the general population. To identify MCS cases in the cohort, we used a delimitation inspired by, but not identical to, the criteria suggested by the 1999 US Consensus Criteria for MCS and the revision suggested by Lacour and colleagues [22,37]. Three criteria have not been included in our delimitation, i.e. “*Are the condition chronic (symptoms been present for at least six months)*?”, “*Do symptoms appear upon being exposed to chemicals in low levels (lower than previously or commonly tolerated)*” and “*Do symptom improve or resolve when the incitants are removed*?” [22,37]. These characteristics of MCS are highly relevant in assessments of MCS patients in clinical settings but were found to be less applicable when screening a large general population-based cohort for participants with an MCS-like phenotype. Nevertheless, it is a possible limitation to the study that results were based on self-reported symptoms and self-reported lifestyle impairments in questionnaires. Although standard epidemiological FSS delimitations were applied, FSS case status was not verified clinically by an experienced physician. This increases the risk of some FSS cases being false positive and findings from this study may not be reproduceable in clinical samples of MCS.

### Conclusions and perspectives

In this study we present in detail the questionnaire tool and algorithm used to delimitate MCS in the DanFund study, as well as selected characteristics of cases with MCS identified in the cohort. We also examined the importance of FSS comorbidity on disease severity. In conclusion, the results suggest that MCS is associated with female sex and low occupational status, low subjective social status, low physical capacity in daily life and poor quality of sleep. MCS cases did not differ from controls with regards to age, education, dietary habits, smoking or weekly alcohol consumption. Moreover, subgroup analyses suggest that the associations between MCS and sedentary lifestyle, physical limitations in daily activities, and poor quality of sleep can almost completely be explained by the presence of FSS comorbidity. Subgroup analyses also revealed that MCS cases with comorbid FSS are more likely to be living alone than both MCS cases with no comorbid FSS and controls. Our finding emphasises the importance of screening MCS cases for FSS comorbidity in future studies of MCS and in clinical assessment of MCS patients.

## Supporting information

S1 Table Prevalence of participants who have experienced unpleasant reactions when exposed to the 11 types of common odours or airborne chemicals. (DOCX)

S2 Table Prevalence of symptoms reported to be associated with inhalation of airborne chemicals. (DOCX)

S3 Table Prevalence of participants who reported adjustment of behaviour due to symptoms related to inhalation of airborne chemicals. (DOCX)

## Abbreviations

CFS: Chronic fatigue syndrome
DanFunD: Danish Study of Functional Disorders
FM: Fibromyalgia
FSS: Functional somatic syndrome
IBS: Irritable bowel syndrome
MCS: Multiple chemical sensitivity
WAD: Whiplash-associated disorders
