# Supplementary material for: Multiple chemical sensitivity described in the Danish general population: Cohort characteristics and the importance of screening for functional somatic syndrome comorbidity—The DanFunD study
Source: PLoS One. 2021 Feb 24;16(2):e0246461. doi: 10.1371/journal.pone.0246461 (PMC7904225; doi:10.1371/journal.pone.0246461)
Supplement: S1 Table — (DOCX) [file pone.0246461.s001.docx]

S1 Table 1: Prevalence of participants who have experienced unpleasant reactions when exposed to these 11 types of common odours or airborne chemicals

| **Have you ever experienced** **unpleasant reactions elicited by inhalation of odours or chemicals from? % (N)** | **MCS all**  **(n=188)** | **MCS + FSS comorbidity**  **(n=73)** | **MCS ÷ FSS comorbidity**  **(n=109)** | **Controls ÷ FSS**  **(n=7791)** |
| --- | --- | --- | --- | --- |
| Other persons wearing of perfume,  aftershave, or deodorant | 90.9 (169)* | 93.1 (67)* | 88.9 (96)* | 29.7 (2298) |
| Motor vehicle exhaust | 82.4 (155)* | 89.0 (65)* | 77.1 (84)* **†** | 33.5 (2591) |
| Cleaning agents | 76.6 (144)* | 76.7 (56)* | 76.1 (83)* | 26.3 (2034) |
| Smoke from wood burner | 66.3 (124)* | 66.7 (48)* | 66.1 (72)* | 22.5 (1740) |
| Nail polish remover, glue, or markers | 61.3 (114)* | 62.5 (45)* | 59.3 (64)* | 19.7 (1522) |
| Freshly printed papers or magazines | 58.6(109)* | 59.2 (42)* | 57.8 (63)* | 18.3 (1413) |
| Cooking fumes | 54.3 (102)* | 60.3 (44)* | 49.5 (54)* | 15.0 (1157) |
| Tar or wet asphalt | 54.1 (100)* | 61.1 (44)* | 49.5 (53)* | 15.5 (1196) |
| New furniture | 53.0 (98)* | 47.9 (34)* | 55.6 (60)* | 12.6 (972) |
| Soft plastic or rubber | 45.7 (85)* | 40.8 (29)* | 47.7 (52)* | 9.1 (707) |
| New electronic equipment | 27.9 (51)* | 22.5 (16)* | 29.2 (31)* | 4.5 (351) |

MCS: Multiple chemical sensitivity; FSS: functional somatic syndromes

MCS all; all participants fulling criteria for MCS. MCS + FSS; participants fulling criteria for MCS and one or more comorbid FSS. MCS**÷** FSS; participants fulling criteria for MCS but no comorbid FSS.

* Pearson Chi-square test comparing MCS groups with controls (p<0.05), adjusted for sex and age.

† Pearson Chi-square test comparing MCS ÷ FSS comorbidity with MCS + FSS comorbidity (p<0.05), adjusted for sex and age.
