# Supplementary material for: Multiple chemical sensitivity described in the Danish general population: Cohort characteristics and the importance of screening for functional somatic syndrome comorbidity—The DanFunD study
Source: PLoS One. 2021 Feb 24;16(2):e0246461. doi: 10.1371/journal.pone.0246461 (PMC7904225; doi:10.1371/journal.pone.0246461)
Supplement: S2 Table — (DOCX) [file pone.0246461.s002.docx]

S1 Table 2: Prevalence of symptoms reported to be associated with inhalation of airborne chemicals

| **Can inhalation of odours**  **or chemicals elicit?** | **MCS all**  **(n=188)** | **MCS + FSS comorbidity**  **(n=73)** | **MCS ÷ FSS comorbidity**  **(n=109)** | **Controls ÷ FSS (n=7791)** |
| --- | --- | --- | --- | --- |
| **Ocular and respiratory symptoms % (n)** |  | | | |
| Nose | 84.0 (158)* | 91.8 (67)* | 78.9 (86)***†** | 39.2 (3056) |
| Eyes | 73.4 (138)* | 80.8 (56)* | 67.0 (73)***†** | 34.6 (2693) |
| Lungs | 66.0 (124)* | 76.7 (56)* | 57.8 (63)***†** | 22.5 (1750) |
| Throat | 50.0 (94)* | 54.8 (40)* | 45.9 (50)* | 16.1 (1253) |
| Mouth | 31.9 (60)* | 37.0 (27)* | 26.6 (29)* | 6.4 (496) |
| Sinuses | 27.7 (52)* | 30.1 (22)* | 24.8 (27)* | 5.0 (388) |
| **Symptoms from the central nervous system % (n)** |  | | | |
| Headache | 80.3 (151)* | 86.3 (63)* | 76.1 (83)* | 31.6 (2459) |
| Difficulties concentrating | 43.6 (82)* | 47.9 (35)* | 39.4 (43)* | 6.4 (497) |
| Dizziness | 39.9 (75)* | 43.8 (32)* | 34.9 (38)* | 9.0 (703) |
| Exhaustion/fatigue | 29.8 (56)* | 38.4 (28)* | 22.9 (25)***†** | 2.9 (228) |
| Grogginess | 19.7 (37)* | 27.4 (20)* | 13.8 (15)***†** | 3.3 (254) |
| Panic/anxiety | 12.2 (23)* | 12.3 (9)* | 10.1 (11)* | 1.5 (116) |
| **Symptoms from other organs % (n)** |  |  |  |  |
| Skin | 48.9 (92)* | 47.9 (35)* | 48.6 (53)* | 11.8 (916) |
| Heart/chest | 17.0 (32)* | 21.6 (16)* | 11.9 (13)***†** | 2.6 (200) |
| Gastrointestinal tract | 14.4 (27)* | 17.8 (13)* | 11.0 (12)* | 2.4 (190) |
| Joints | 10.1 (19)* | 13.7 (10)* | 6.4 (7)* | 1.2 (96) |
| Muscles | 9.6 (18)* | 12.3 (9)* | 6.4 (7)* | 1.0 (80) |
| Urinary tract | 4.3 (8)* | 5.5 (4)* | 1.8 (2) | 0.7 (56) |

Multiple chemical sensitivity (MCS), functional somatic syndrome (FSS)

MCS all; all participants fulling criteria for MCS. MCS + FSS; participants fulling criteria for MCS and one or more comorbid FSS. MCS ÷ FSS; participants fulling criteria for MCS but no comorbid FSS.

*Pearson Chi-square test comparing MCS groups with controls (p<0.05), adjusted for sex and age.

† Pearson Chi-square test comparing MCS ÷ FSS comorbidity with MCS + FSS comorbidity (p<0.05), adjusted for sex and age.
