# Supplementary material for: Multiple chemical sensitivity described in the Danish general population: Cohort characteristics and the importance of screening for functional somatic syndrome comorbidity—The DanFunD study
Source: PLoS One. 2021 Feb 24;16(2):e0246461. doi: 10.1371/journal.pone.0246461 (PMC7904225; doi:10.1371/journal.pone.0246461)
Supplement: S3 Table — (DOCX) [file pone.0246461.s003.docx]

S1 Table 3: Prevalence of participants who reported adjustment of behaviour due to symptoms related to inhalation of airborne chemicals

| **Has unpleasant reactions elicited by inhalation of odours or chemicals lead to:** | **MCS all**  **(n=188)** | **MCS + FSS comorbidity**  **(n=73)** | **MCS ÷ FSS comorbidity**  **(n=109)** | **Controls ÷ FSS (n=7791)** |
| --- | --- | --- | --- | --- |
| **Adjustments of personal lifestyle? % (n)** |  | | | |
| Personal hygiene products | 89.9 (169)* | 86.3 (63)* | 91.7 (100)* | 38.9 (2968) |
| Cleaning at home | 80.3 (147)* | 78.6 (55)* | 82.4 (89)* | 24.5 (1859) |
| Choice of shopping places | 53.9 (97)* | 52.2 (36)* | 47.8 (33)* | 10.4 (786) |
| **Adjustments of social life? % (n)** |  | | | |
| Public transportation | 55.1 (102)* | 62.9 (44)* | 52.3 (57)* | 0.6 (47) |
| Gatherings in the public sphere | 31.4 (58)* | 34.3 (24)* | 28.4 (31)* | 0.3 (21) |
| Social functions in the private sphere | 29.0 (54)* | 28.2 (20)* | 29.4 (32)* | 0.3 (23) |
| **Adjustments** **of occupational conditions? % (n)** |  | | | |
| Sick leave from work or school | 31.9 (59)* | 32.4 (23)* | 31.5 (34)* | 0.3 (25) |
| Left employment or education permanently | 14.8 (27)* | 12.7 (9)* | 16.0 (17)* | 0.1 (11) |
| Inability to work or study | 9.3 (17)* | 11.3 (8)* | 7.5 (8)* | 0.0 (3) |

Multiple chemical sensitivity (MCS), functional somatic syndrome (FSS)

MCS all; all participants fulling criteria for MCS. MCS + FSS; participants fulling criteria for MCS and one or more comorbid FSS. MCS ÷ FSS; participants fulling criteria for MCS but no comorbid FSS.

*Pearson Chi-square test comparing MCS groups with controls (p<0.05), adjusted for sex and age.
